# Supplementary material for: Gut Microbial Catabolites of Tryptophan Are Ligands and Agonists of the Aryl Hydrocarbon Receptor: A Detailed Characterization
Source: Int J Mol Sci. 2020 Apr 9;21(7):2614. doi: 10.3390/ijms21072614 (PMC7177849; doi:10.3390/ijms21072614)
Supplement: Supplementary file 1 [file ijms-21-02614-s001.pdf]

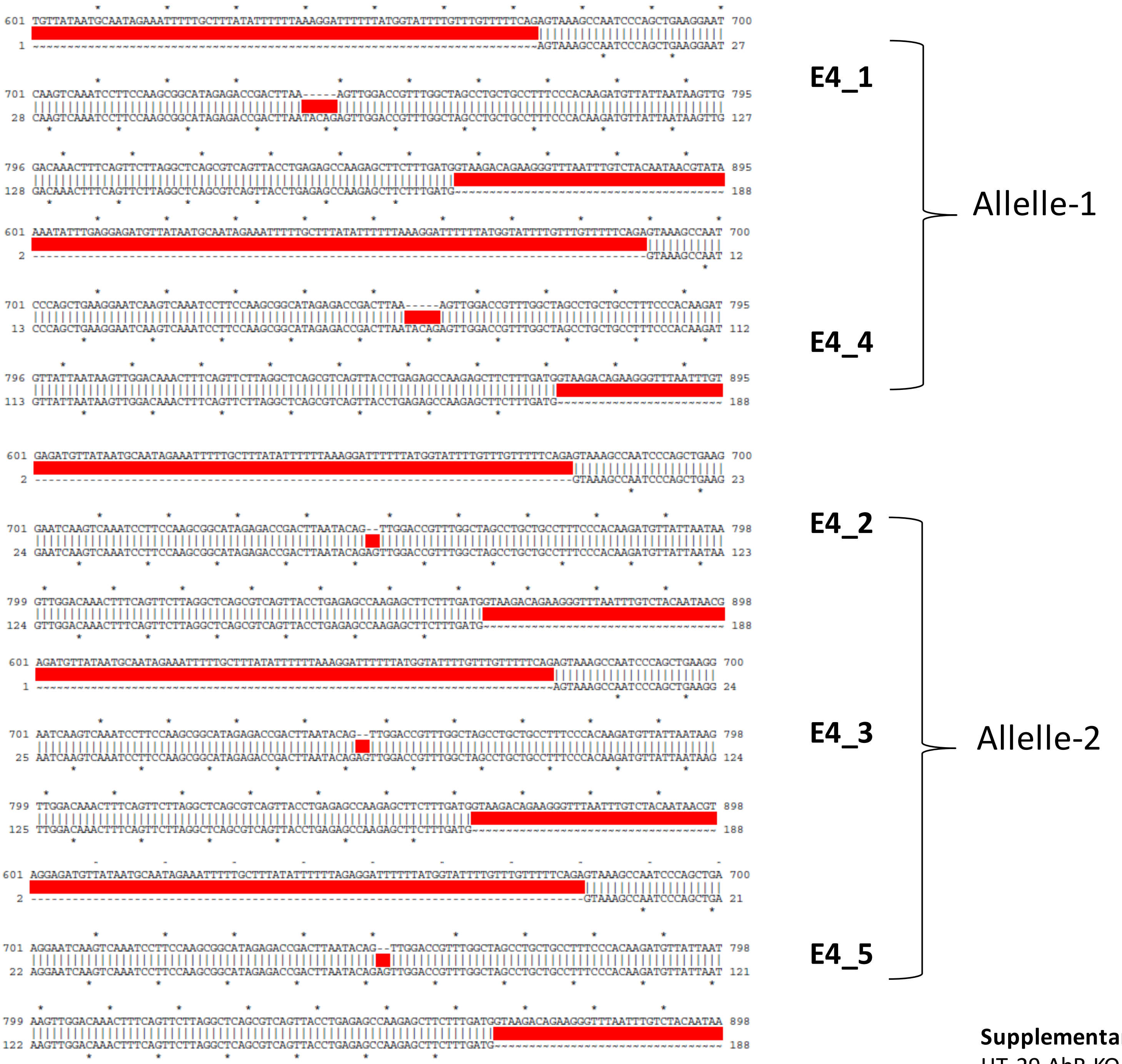


**Supplementary Figure 1.** Sequencing data of HT-29 AhR KO clone E4 variant.

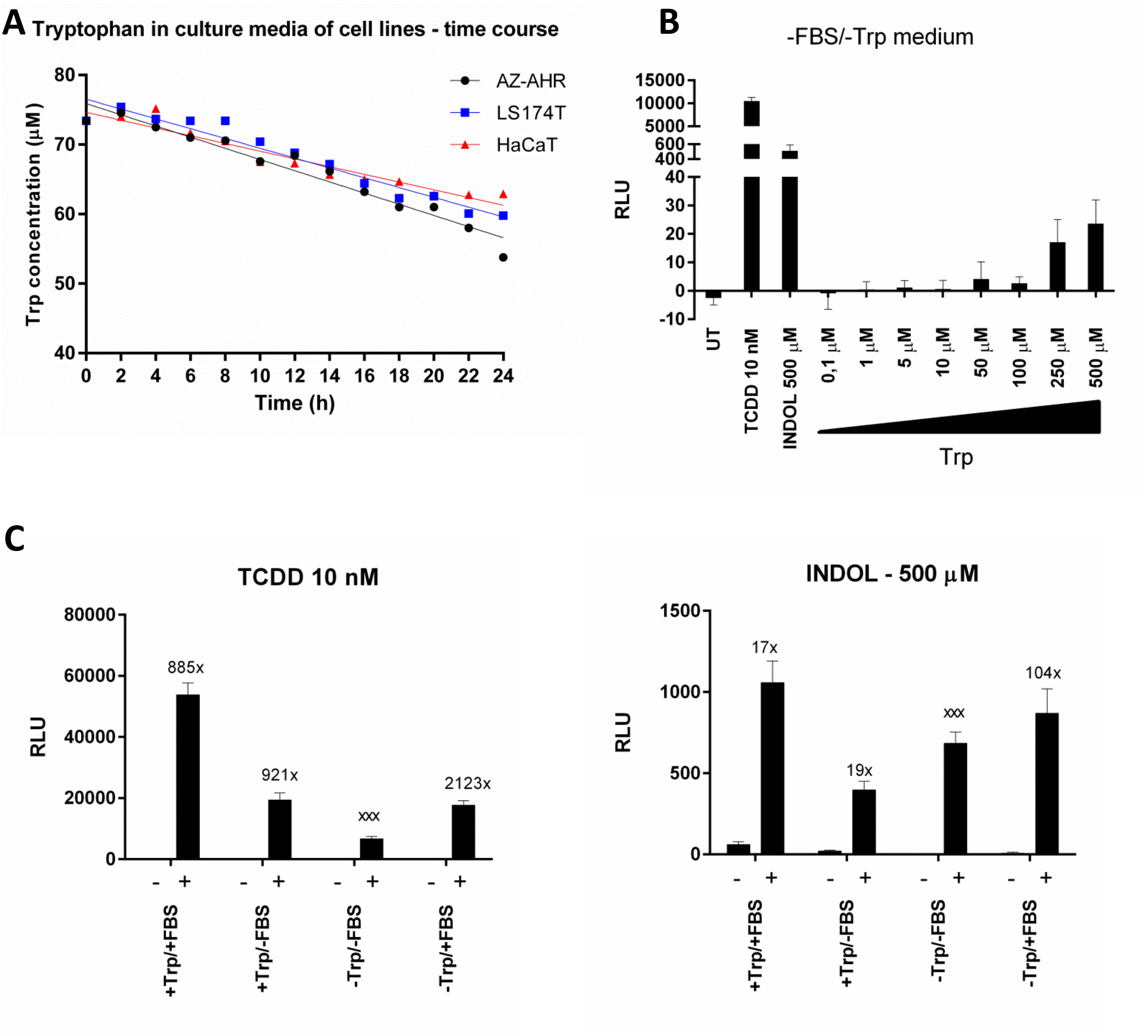


**Supplementary Figure 2. Tryptophan effects in culture medium on AhR activity.** (A) Time-course of Trp levels in culture media from hepatic (AZ-AHR), instestinal (LS174T) and skin (HaCaT) cells. Determined by HPLC. (B) Induction of AhR-dependent luciferase activity in AZ-AHR cells incubated for 24 h with TCDD, Indole and Trp in Trp/serum-free culture medium. (C) Induction of AhR-dependent luciferase activity in AZ-AHR cells incubated for 24 h with TCDD and Indole in standard (+Trp/+FBS), serum-free (+Trp/-FBS), Trp-free (-Trp/+FBS) and Trp/serum-free (-Trp/-FBS) culture medium.

| incubation | | # fields | ∑ cells | # positive AhR nuclei | % positive AhR nuclei | % relative AhR nuclei | Ø AhR positive nuclei |
|---|---|---|---|---|---|---|---|
| Vehicle (DMSO) | exp 1 | 4 | 316 | 17 | 5 | 9 | 13 ± 11.6 |
| | exp 2 | 11 | 545 | 19 | 3 | 4 | |
| | exp 3 | 11 | 553 | 90 | 16 | 26 | |
| 10 nM TCDD | exp 1 | 7 | 318 | 171 | 54 | 100 | 100 ± 11.8 |
| | exp 2 | 14 | 555 | 430 | 77 | 100 | |
| | exp 3 | 11 | 538 | 330 | 61 | 100 | |
| 1 mM Indole | exp 1 | 6 | 335 | 203 | 61 | 113 | 105 ± 9.2 |
| | exp 2 | 9 | 338 | 248 | 73 | 95 | |
| | exp 3 | 12 | 500 | 323 | 65 | 107 | |
| 200 µM IPY | exp 1 | 7 | 326 | 136 | 42 | 78 | 73 ± 9.0 |
| | exp 2 | 9 | 295 | 176 | 60 | 78 | |
| | exp 3 | 11 | 514 | 194 | 38 | 62 | |
| 200 µM 3-MI | exp 1 | 6 | 407 | 264 | 65 | 120 | 105 ± 13.8 |
| | exp 2 | 9 | 329 | 236 | 72 | 94 | |
| | exp 3 | 13 | 603 | 375 | 62 | 102 | |
| 200 µM TA | exp 1 | 6 | 421 | 187 | 44 | 81 | 91 ± 14.6 |
| | exp 2 | 11 | 319 | 208 | 65 | 84 | |
| | exp 3 | 12 | 527 | 346 | 66 | 108 | |
| 100 µM IAC | exp 1 | 6 | 393 | 199 | 51 | 94 | 98 ± 20.4 |
| | exp 2 | 9 | 356 | 218 | 61 | 79 | |
| | exp 3 | 11 | 499 | 366 | 73 | 120 | |
| 200 µM IAD | exp 1 | 7 | 377 | 175 | 46 | 85 | 83 ± 13.1 |
| | exp 2 | 9 | 344 | 252 | 73 | 95 | |
| | exp 3 | 11 | 538 | 228 | 42 | 69 | |

**Suppl. Table 1. Nuclear translocation of AhR – quantification of immunofluorescence.**
LS180 cells were incubated for 90 min with MICT, vehicle or TCDD. Intracellular AhR was visualized with Alexa Fluor 488 labelled primary antibody. The whole staining protocol was performed in three independent experiments in technical duplicates. The AhR translocation was evaluated visually depending on the distinct signal intensity of AhR antibody in the nucleus and cytosol. For percentage calculation, approximately one hundred cells from at least four randomly selected fields of view in each replicate were used.
